# Supplementary material for: Analyses of Genes Critical to Tumor Survival Reveal Potential ‘Supertargets’: Focus on Transcription
Source: Cancers (Basel). 2023 Jun 3;15(11):3042. doi: 10.3390/cancers15113042 (PMC10252933; doi:10.3390/cancers15113042)
Supplement: Supplementary file 1 [file cancers-15-03042-s001.zip › cancers-2405642-Supplementary File S1. Supertargets tables.pdf]

Top-5 Supertargets for 27 cancer types presented in DepMap

Med-all, median expression of the Supertargets in all tumor cell lines.

Med-CS (cancer specific), median expression of the Supertargets in tumor cells in which it was identified.

Blood malignancies

Acute lymphocytic leukemia

Gene	T-statistics	P-value	Function	Domain	Expression	PubMed entries
ATP6V0A2	-14.5	1.12E-43	Part of the proton channel of V-ATPases	V-type ATPase	Med all 3.52<br>Med CS 4.3	70
MYB	-13.3	1.84E-37	Transcription factor	HTH DNA-binding domain	Med all 2.08<br>Med CS **7.4**	7 570
LEF1	-13.3	2.71E-37	Transcription factor	HMG box DNA-binding domain	Med all 2.37<br>Med CS **8.83**	1 520
RUNX1	-13.2	5.29E-37	Transcription factor	HTH-type DNA-binding domain	Med all 3.57<br>Med CS 5.88	4 378
EBF1	-12.2	3.6E-32	Transcription factor	HLH DNA-binding domain	Med all 0.9<br>Med CS **5.88**	547

Acute myelogenous leukemia

Gene	T-statistics	P-value	Function	Domain	Expression	PubMed entries
MYB	-20.1	6.73E-76	Transcription factor	HTH DNA binding domain	Med all 2.08<br>Med CS **7.11**	7 570

SPI1	-18.1	6.9E-64	Transcription factor	HTH-type DNA binding domain	Med all 0.68<br>Med CS **6.89**	608
LMO2	-17.2	1.44E-58	Member of multiprotein DNA binding complex	2 LIM domain	Med all 1.33<br>Med CS **6.49**	606
JAK2	-14.6	3.04E-44	Non-membrane spanning tyrosine kinase activity	B41, SH2, tyrosine kinase domain	Med all 1.73<br>Med CS 2.39	11 972
GFI1	-13.9	4.04E-40	Transcription factor	6 zinc fingers of C2H2-type	Med all 1.19<br>Med CS **5.41**	460

Chronic myelogenous leukemia

**Gene**	**T-statistics**	**P-value**	**Function**	**Domain**	**Expression**	**PubMed entries**
BCR	-30	1.48E-142	Serine/threonine kinase activity; the part of the BCR-ABL fusion protein	RhoGEF, PH, C2, RhoGAP	Med all 3.95<br>Med CS 4.7	24 546
ABL1	-23.5	2.89E-98	Tyrosine kinase, the part of the BCR-ABL fusion protein	SH3, SH2, TyrKc, FABD	Med all 4.62<br>Med CS 5.18	3 034
GAB2	-16.5	3.16E-54	Adapter protein	PH	Med all 2.53<br>Med CS **5.11**	484
SOS1	-16.3	3.92E-53	Guanine nucleotide exchange factor for Ras proteins	RhoGEF, PH, RasGEFN, RasGEF	Med all 3.34<br>Med CS 3.66	1 009
STAT5B	-15.8	1.08E-50	Signal transduction and activation of transcription	DNA binding domain with an immunoglobulin-like fold, SH2	Med all 4.13<br>Med CS 5.96	1 223

Hodgkin's lymphoma

Gene	T-statistics	P-value	Function	Domain	Expression	PubMed entries
IL13RA1	-21.6	1.36E-85	Cytokine-mediated signaling	IgG-like, cytokine-binding domain, fibronectin type III	Med all 4.18 Med CS 4.9	357
STAT6	-15.5	1.25E-48	Signal transduction, transcriptional activation	STAT DNA binding, SH2	Med all 5.3 Med CS 6.87	3 531
IL4R	-9.63	4.42E-21	Receptor for interleukins 4 and 13	Fibronectin type-III	Med all 3.76 Med CS 6.11	436
IKZF1	-8.26	4.53E-16	Transcription factor	6 tandemly placed C2H2-type zinc fingers	Med all 0.98 Med CS 6.59	921
BATF3	-6.14	1.15E-09	Transcription factor	bZIP	Med all 1.65 Med CS 8.2	270

Non-Hodgkin lymphoma

Gene	T-statistics	P-value	Function	Domain	Expression	PubMed entries
MEF2B	-21.4	2.2E-84	Transcription factor	MADS DNA binding domain	No data	121
EBF1	-19.4	8.16E-72	Transcription factor	IPT and HLH DNA binding domain	Med all 0.9 Med CS 1.92	554
BCL6	-17.6	6.77E-61	Transcription factor	BTB/POZ domain, 6 tandemly placed C2H2-	Med all 3.18 Med CS 2.93	3 694

				type Zinc fingers		
SH3GL1	-16.5	2.4E-54	Endocytosis; a role in the cell cycle	BAR, SH3	Med all 5.3<br>Med CS 4.38	42
PAX5	-16.5	3.12E-54	Transcription factor	PAX DNA binding domain	Med all 0.68<br>Med CS 3.65	1 952

Plasma cell/multiple myeloma

Gene	T-statistics	P-value	Function	Domain	Expression	PubMed entries
IRF4	-24.8	1.56E-106	Transcription factor	IRF (tryptophan pentad repeat) DNA binding domain, IRF-3	No data	1 487
PRDM1	-22.7	5.93E-93	Transcription factor	SET, 5 tandemly placed C2H2-type zinc fingers	No data	1 021
POU2AF1	-19.3	3.51E-71	Transcription factor	POU DNA binding domain	No data	204
HERPUD1	-18.7	1.04E-67	Endoplasmic reticulum quality control system	Ubiquitin-like	No data	130
MEF2C	-18.3	3.39E-65	Transcription factor	MADS DNA binding domain	No data	1 372

Solid tumors

Bile duct

Gene	T-statistics	P-value	Function	Domain	Expression	PubMed entries
DOCK5	-6,74	2,64E-11	Guanine nucleotide exchange factors for small Rho family G proteins	SH3, C2 DOCK-type, DOCKER	Med all 2.83 Med CS 3.43	62
EGFR	-6,64	5,15E-11	Transmembrane glycoprotein, receptor for EGF family members.	FU, tyrosine kinase catalytic domain	Med all 3.49 Med CS 5.34	106 644
TRPM7	-5,45	6,23E-08	Ion channel and serine/threonine protein kinase	Alpha-type protein kinase	Med all 3.89 Med CS 3.77	823
ARHGEF7	-5,33	1,20E-07	Ras-like family of Rho proteins	CH,SH3, RhoGEF, PH	Med all 4.45 Med CS 3.95	126
ABCF1	-5,13	3.45E-07	Member of the superfamily of ATP-binding cassette transporters.	ABC transporter	Med all 5.58 Med CS 5.1	53

Bone - Ewing sarcoma

Gene	T-statistics	P-value	Function	Domain	Expression	PubMed entries
TRIM8	-23.2	5.34E-96	E3 ubiquitin-protein ligase	RING	Med all 4,82 Med CS 3,87	67
FLI1	-19	1.92E-69	Transcription factor, part of the EWS-FLI1 chimeric protein	SAM PNT-type, ETS DNA binding domain	Med all 1,63 Med CS **4,72**	2 004

			characteristic for Ewing sarcoma	(variation of the HLH domain)		
ETV6	-14.1	2.39E-41	Transcription factor involved in chromosomal rearrangements associated with leukemia and congenital fibrosarcoma	SAM PNT-type, ETS DNA-binding domain (variation of the HLH domain)	Med all 2.73 Med CS 2.04	1 527
IGF2BP1	-12.3	1.7E-32	RNA binding, regulation of translation	2 RRM domain, 4 KH domain	Med all 1.78 Med CS **5.92**	391
STAG1	-10.6	6.29E-25	Component of cohesin complex	SCD	Med all 3.64 Med CS 4.08	83

Bone - osteosarcoma

**Gene**	**T-statistics**	**P-value**	**Function**	**Domain**	**Expression**	**PubMed entries**
SMARCAL1	-5.51	4.5E-08	Stabilization of damaged replication forks	DEAD-like helicase, helicase superfamily c-terminal domain	Med all 3.64 Med CS 3.78	162
IRS1	-5.16	2.95E-07	Insulin regulated processes by insulin	PH, Phosphotyrosine-binding IRS domain	Med all 2.52 Med CS 3.17	6 093
SUB1	-5.16	2.99E-07	General coactivator of transcription	PC4 transcriptional coactivator domain	Med all 7.43 Med CS 7.62	414
HMGA2	-5.11	3.82E-07	Transcription factor	AT-hook HMG box DNA-binding domain	Med all 3.41 Med CS 2.58	1 607
FANCM	-5.06	4.85E-07	DNA dependent ATPase component of the Fanconi	DEAD-like helicase, helicase superfamily c-terminal domain	Med all 2.45 Med CS 2.7	273

			anemia core complex			

Breast

Gene	T-statistics	P-value	Function	Domain	Expression	PubMed entries
SPDEF	-17.5	3.6E-60	Transcription factor	SAM PNT-type, ETS DNA-binding domain (variation of the HLH domain)	Med all 0.73 Med CS **5.97**	219
FOXA1	-14.4	5.14E-43	Transcription factor	FORKHEAD DNA-binding domain	Med all 1.88 Med CS **5.03**	1 330
TRPS1	-14.4	5.54E-43	Transcription factor	9 non-tandem C2H2 type zinc fingers, zinc finger_GATA type DNA-binding domain	Med all 1.83 Med CS **4.78**	269
ESR1	-11.5	5.33E-29	Transcription factor	Ligand binding domain, DNA binding zinc finger_C4 domain	Med all 0.41 Med CS **1.22**	5 797
GATA3	-8.78	6.52E-18	Transcription factor	two DNA binding GATA-type zinc fingers	Med all 1.72 Med CS **4.91**	4 524

Glioma

Gene	T-statistics	P-value	Function	Domain	Expression	PubMed entries
PRKAR1A	-11.6	2.21E-29	Regulatory subunit of cAMP-dependent protein kinases	RIIa, cNMP	Med all 6,56 Med CS 6,97	692
JUN	-10	1.22E-22	Transcription factor	bZIP	Med all 4,99 Med CS 6,77	3 725

RPP25L	-10	1.24E-22	May be a component of ribonuclease P	Alba-like DNA/RNA-binding domain	Med all 4,67 Med CS 4,42	0
LMNA	-9.7	2.48E-21	Component of the nuclear lamina	IF	Med all 8,5 Med CS 8,84	1 839
VRK1	-9.25	1.24E-19	Serine/threonine kinase in cell cycle regulation	Protein kinase domain	Med all 5,1 Med CS 4,77	164

Cervix

Gene	T-statistics	P-value	Function	Domain	Expression	Pub Med entries
ZER1	-15.7	8.98E-50	Subunit of E3 ubiquitin ligase complex	-	Med all 3,76 Med CS 3,53	9*
UBE3A	-13.2	8.08E-37	E3 ubiquitin ligase	HECT	Med all 4,88 Med CS 4,88	888
SPTY2D1	-6.25	6.1E-10	Histone chaperone	SPT2	Med all 3.43 Med CS 3.34	7
CRTC2	-6.15	1.08E-09	Transcriptional coactivator for CREB1	TORC N,M and C	Med all 4.68 Med CS 4.27	441
NFIA	-5.7	1.56E-08	Transcription factor	CTF /nuclear factor 1 DNA binding domain	Med all 1.87 Med CS 2.72	276

*(62 for CRL2 synonym)

Colorectal

Gene	T-statistics	P-value	Function	Domain	Expression	PubMed entries
CTNNB1	-20.7	4.25E-80	Key downstream component of Wnt pathway	12 ARM domain	Med all 6,84<br>Med CS 7,1	9 797
TCF7L2	-18.4	2.16E-65	Transcription factor	HMG box DNA binding domain	Med all 3,14<br>Med CS 4,11	1 779
CDX2	-15.2	3.71E-47	Transcription factor	Homeodomain	Med all 0,72<br>Med CS **5,56**	2 934
KLF5	-11.9	6.95E-31	Transcription factor	3 tandemly placed C2H2-type zinc fingers	Med all 3,53<br>Med CS 6,41	723
SATB2	-10.6	4.75E-25	Transcription factor	Two CUTs and one homeodomain<br>DNA binding domain	Med all 2,01<br>Med CS 2,91	579

Esophagus

Gene	T-statistics	P-value	Function	Domain	Expression	PubMed entries
FIS1	-6.18	9.31E-10	Control of mitochondrial morphology; cytochrome c release	Fis1	Med all 6.72<br>Med CS 6.52	638
TIMM17A	-5.98	3.03E-09	Translocation of transit peptide-containing proteins across the mitochondrial inner membrane	-	Med all 6.2<br>Med CS 6.13	15

TRMT10A	-5.98	3.09E-09	tRNA methyltransferase	TRM10-type	Med all 6.19<br>Med CS 6.13	13
ADAR	-5.95	3.62E-09	RNA specific adenosine deaminase	DSRM, ADEAM	Med all 6,1<br>Med CS 6,16	1 653
ERBB3	-5.7	1.56E-08	Member of the EGFR family of receptor tyrosine kinases	FU, TyrKc	Med all 3,01<br>Med CS 5,12	2 509

Stomach

Gene	T-statistics	P-value	Function	Domain	Expression	PubMed entries
FAM126B	-7.19	1.22E-12	Localization of phosphatidylinositol 4-kinase (PI4K) to the plasma membrane	-	Med all 2.23<br>Med CS 2.26	4
TCF7L2	-6.76	2.35E-11	Transcription factor	HMG box DNA-binding domain	Med all 3.14<br>Med CS 4.43	1 779
CTNNB1	-5.97	3.33E-09	Key downstream component of the canonical Wnt signaling pathway	12 ARM domain	Med all 6.84<br>Med CS 7.25	9 797
RAB10	-5.77	1.06E-08	Member of the RAS superfamily of small GTPases.	Ras	Med all 6,19<br>Med CS 6,4	271
GATA4	-5.46	6.12E-08	Transcription factor	2 DNA-binding GATA-type zinc fingers	Med all 0,94<br>Med CS **2,91**	3 002

Kidney

Gene	T-statistics	P-value	Function	Domain	Expression	PubMed entries
PAX8	-14.8	2.41E-45	Transcription factor	PAX DNA-binding domain	Med all 1,86<br>Med CS **6,36**	1 833
HNF1B	-13.9	1.52E-40	Transcription factor	Homeodomain	Med all 0,89<br>Med CS **5,5**	1 205
SEC23IP	-10	1.56E-22	Organization of endoplasmic reticulum exit sites.	SAM, DDHD	Med all 4,04<br>Med CS 4,12	23
EPAS1	-9.59	6.48E-21	Transcription factor	PAS, PAC; HLH DNA-binding domain	Med all 3,95<br>Med CS 4,74	465
PARD3	-9	1.03E-18	Adapter protein involved in asymmetrical cell division and polarization	3 PDZ domain	Med all 4,26<br>Med CS 5,78	343

Liver

Gene	T-statistics	P-value	Function	Domain	Expression	PubMed entries
HS2ST1	-5.84	7.01E-09	Heparan sulfate biosynthesis	-	Med all 3,93<br>Med CS 4,35	19
FGFR1	-5.61	2.53E-08	Receptor tyrosine kinase for fibroblast growth factors	Immunoglobulin C-2 Type, TyrKc	Med all 3,9<br>Med CS 5,12	4 732
HNF4A	-5.58	3.08E-08	Transcription factor	HOLI, DNA-binding C4-type zinc fingers	Med all 0,6<br>Med CS **1,99**	1 583
KLB	-5.39	8.85E-08	Binding of fibroblast growth factor 21 to the cognate receptor	-	Med all 0,14<br>Med CS **0,69**	413

NDST1	-5.3	1.39E-07	Catalyzes both the N-deacetylation and the N-sulfation of glucosamine (GlcNAc) of the glycosaminoglycan in heparan sulfate	Heparan sulphate-N-deacetylase, sulfotransferase domain	Med all 4,32<br>Med CS 5,07	127

Lung

Gene	T-statistics	P-value	Function	Domain	Expression	PubMed entries
NKX2-1	-8.61	2.75E-17	Transcription factor	Homeodomain	Med all 0,65<br>Med CS **1,17**	834
NFE2L2(NRF2)	-7.86	9.66E-15	Transcription factor	bZIP	Med all 5,82<br>Med CS 6,04	8 359
SMARCA2	-7.1	2.38E-12	Component of SWI/SNF-chromatin remodeling complex	QLQ, HAS, BRK, DEXDc, HELICc, SnAC, BROMO	Med all 4,59<br>Med CS 4,45	551
DDX5	-5.94	3.97E-09	ATP-dependent RNA helicase (RNA stability/ splicing)	DEXDc, HELICc	Med all 8,38<br>Med CS 8,44	350
NRDC	-5.83	7.62E-09	Peptide cleavage at the N-terminus of Arg residues in dibasic pairs	Peptidase M16	Med all 6,35<br>Med CS 6,64	97

Ovary

Gene	T-statistics	P-value	Function	Domain	Expression	PubMed entries
PAX8	-13.6	6.13E-39	Transcription factor	PAX DNA-binding domain	Med all 1.86 Med CS **6.4**	1 833
PARD3	-9.31	7.39E-20	Adapter protein involved in asymmetrical cell division and polarization	Three PDZ domain	Med all 4.26 Med CS 5.05	343
SOX17	-9.16	2.83E-19	Transcription factor	HMG box DNA binding domain	Med all **0.32** Med CS 3.68	869
WT1	-8.67	1.61E-17	Transcription factor	Four tandemly placed C2H2-type zinc fingers	Med all 1.06 Med CS 1.16	6 581
CCNE1	-8.4	1.44E-16	G1/S transition control	CYCLIN, Cyclin_c	Med all 3.63 Med CS 4.39	877

Pancreas

Gene	T-statistics	P-value	Function	Domain	Expression	PubMed entries
KRAS	-15	4.15E-46	Member of the RAS superfamily of small GTPases, regulation of cell proliferation	Ras	Med all 4,03 Med CS 4,13	21 810
DOCK5	-12.8	5.01E-35	Guanine nucleotide exchange factor for Rho and Rac.	SH3, C2 DOCK-type, DOCKER	Med all 2,83 Med CS 3,87	63 (0)
ID1	-8.4	1.43E-16	Transcriptional regulator devoid of DNA binding activity	HLH	Med all 5,75 Med CS 6,98	1 809
FZD5	-8.26	4.36E-16	Receptor for Wnt proteins	FRI, Frizzled	Med all 1,74 Med CS 2,17	208

WLS	-7.31	5.46E-13	Sorting and secretion of Wnt proteins	-	Med all 4,47<br>Med CS 5,26	726

Neuroblastoma

Gene	T-statistics	P-value	Function	Domain	Expression	PubMed entries
ISL1	-24.6	1.53E-105	Transcription factor	2 LIM domain and homeodomain	Med all 0.83<br>Med CS **4,85**	970
HAND2	-22	1.84E-88	Transcription factor	HLH DNA-binding domain	Med all 0,7<br>Med CS **8.2**	548
PHOX2B	-16.8	2.17E-56	Transcription factor	Homeodomain	Med all 0,17<br>Med CS **6,6**	659
PHOX2A	-16.3	2.32E-53	Transcription factor	Homeodomain	Med all 0.22<br>Med CS **7.3**	199
MYCN	-13.7	1.75E-39	Transcription factor	HLH DNA binding domain	Med all 0.87<br>Med CS **9.44**	2 871

Skin (squamous cell carcinoma, Merkel cell carcinoma, melanoma)

Gene	T-statistics	P-value	Function	Domain	Expression	PubMed entries
SOX10	-32.6	7.93E-161	Transcription factor	HMG box DNA-binding domain	Med all 0.58<br>Med CS **7,59**	1 884
BRAF	-25.9	3.99E-114	Protein kinase that phosphorylates MAP2K1 and	RBD, C1	Med all 3.1<br>Med CS 3.17	19 157

			activates the MAP kinase signaling pathway			
MITF	-21	5.56E-82	Transcription factor	HLH DNA-binding domain	Med all 2 Med CS **5.79**	2 923
DUSP4	-18.9	2.04E-68	Regulates mitogenic signal transduction by dephosphorylating Thr and Tyr residues on ERK1 and ERK2.	RHOD, DSPc	Med all 3.05 Med CS **6.55**	239
MAPK1	-18.7	1.77E-67	MAP protein kinase	S_TKc	Med all 5.05 Med CS 5.48	3 205

Soft tissues - liposarcoma

Gene	T-statistics	P-value	Function	Domain	Expression	PubMed entries
CPM	-14.6	3.49E-44	Specific removal of C-terminal basic residues Arg or Lys from peptides	Zinc_pept	Med all 1,86 Med CS **5,71**	6 999
BEST3	-11.2	1.16E-27	Ca^{2+}-sensitive Cl channel formation	-	Med all 0,67 Med CS **2,5**	34
SEC23A	-9.31	7.77E-20	Component of the coat protein complex II (COPII)	Gelsolin-like, ZincF Sec23/Sec24-type	Med all 5.07 Med CS 6.11	117
HMGA2	-9.16	2.77E-19	Transcription factor	AT-hook HMG box DNA binding domain	Med all 3.41 Med CS 5.62	1 613
CAPS2	-8.97	1.34E-18	Calcium-binding protein involved in exocytosis of vesicles filled with neurotransmitters and neuropeptides	C2, PH, DUF1041	Med all 1.6 Med CS 1,45	760

Soft tissues – rhabdomyosarcoma

Gene	T-statistics	P-value	Function	Domain	Expression	PubMed entries
MYOD1	-29.7	3,00E-140	Transcription factor	HLH DNA binding domain	Med all 0,11 Med CS **5,6**	1 479
PAX3	-23	4.82E-95	Transcription factor	PAX DNA binding domain	Med all 0,59 Med CS **2,51**	1 975
MYOG	-16.1	2.11E-52	Transcription factor	HLH DNA binding domain	Med all 0,11 Med CS **5,67**	1 312
ZBTB18	-11.6	2.65E-29	Transcription factor	BTB, Four tandemly placed C2H2-type zinc fingers	Med all 3,09 Med CS 3,85	52
DLL1	-10.3	6.09E-24	Transmembrane ligand protein of Notch1-3 receptors	DSL, 8 EGF-like domain	Med all 1,07 Med CS 2,04	606

Upper aerodigestive

Gene	T-statistics	P-value	Function	Domain	Expression	PubMed entries
TP63	-16.3	2.12E-53	Transcription factor	P53 DNA-binding domain, SAM domain	No data	2 2020
EGFR	-10.2	2.31E-23	Transmembrane glycoprotein, receptor for EGF family proteins	FU, tyrosine kinase catalytic domain	No data	106 644
ITGB1	-9.7	2.26E-21	Receptors for collagen (cell adhesion)	INB, EGF-like, Integrin	No data	642

EDF1	-9.41	3.04E-20	Transcriptional coactivator	Helix-turn-helix XRE family-like	No data	51
ATP6V0E1	-9	1,00E-18	Vacuolar ATPase (acidification of intracellular compartments)	-	No data	6

Urinary tract

Gene	T-statistics	P-value	Function	Domain	Expression	PubMed entries
PPARG	-9.56	7.8E-21	Member of the peroxisome proliferator-activated nuclear receptors	C4-type zinc finger, HOLI	Med all 2,6<br>Med CS 0,8	2 253
SOX4	-7.37	3.38E-13	Transcription factor	HMG box DNA binding domain	Med all 4,04<br>Med CS 4,51	756
RXRA	-6.73	2.86E-11	Transcription factor	C4-type zinc finger, HOLI	Med all 3.51<br>Med CS 3.35	330
TEAD3	-5.75	-5.75	Transcription factor	TEA DNA-binding domain	Med all 3.12<br>Med CS 3.5	56
SNAP29	-5.54	3.86E-08	Receptor of N-ethylmaleimide-sensitive factor-attachment protein	t_SNARE	Med all 4.11<br>Med CS 4.01	141

Uterus

Gene	T-statistics	P-value	Function	Domain	Expression	PubMed entries
CCNE1	-7.89	7.75E-15	Control of the cell cycle at the G1/S transition.	CYCLIN, Cyclin_c	Med all 3.63<br>Med CS 4.57	880
PAX8	-7.47	1.65E-13	Transcription factor	PAX DNA-binding domain	Med all 1,86<br>Med CS **6,6**	1 839
WRN	-6.63	5.23E-11	DNA helicase that involves in DNA repair, replication, transcription and telomere maintenance	35EXOc, DEXDc, HELIC, RQC, HRDC	Med all 3.15<br>Med CS 3.35	1 238
MECOM	-5.63	2.3E-08	Histone-lysine N-methyltransferase	SET, 10 C2H2-type zinc fingers placed tandemly	Med all 2.01<br>Med CS 2.39	561
WDR88	-5.37	9.82E-08	Unknown	PQQ, 6 WD40 repeats	Med all 0.2<br>Med CS 0.21	1
