# Supplementary material for: Analyses of Genes Critical to Tumor Survival Reveal Potential ‘Supertargets’: Focus on Transcription
Source: Cancers (Basel). 2023 Jun 3;15(11):3042. doi: 10.3390/cancers15113042 (PMC10252933; doi:10.3390/cancers15113042)
Supplement: Supplementary file 1 [file cancers-15-03042-s001.zip › cancers-2405642-Supplementary File S2. Supplementary Figures.pdf]

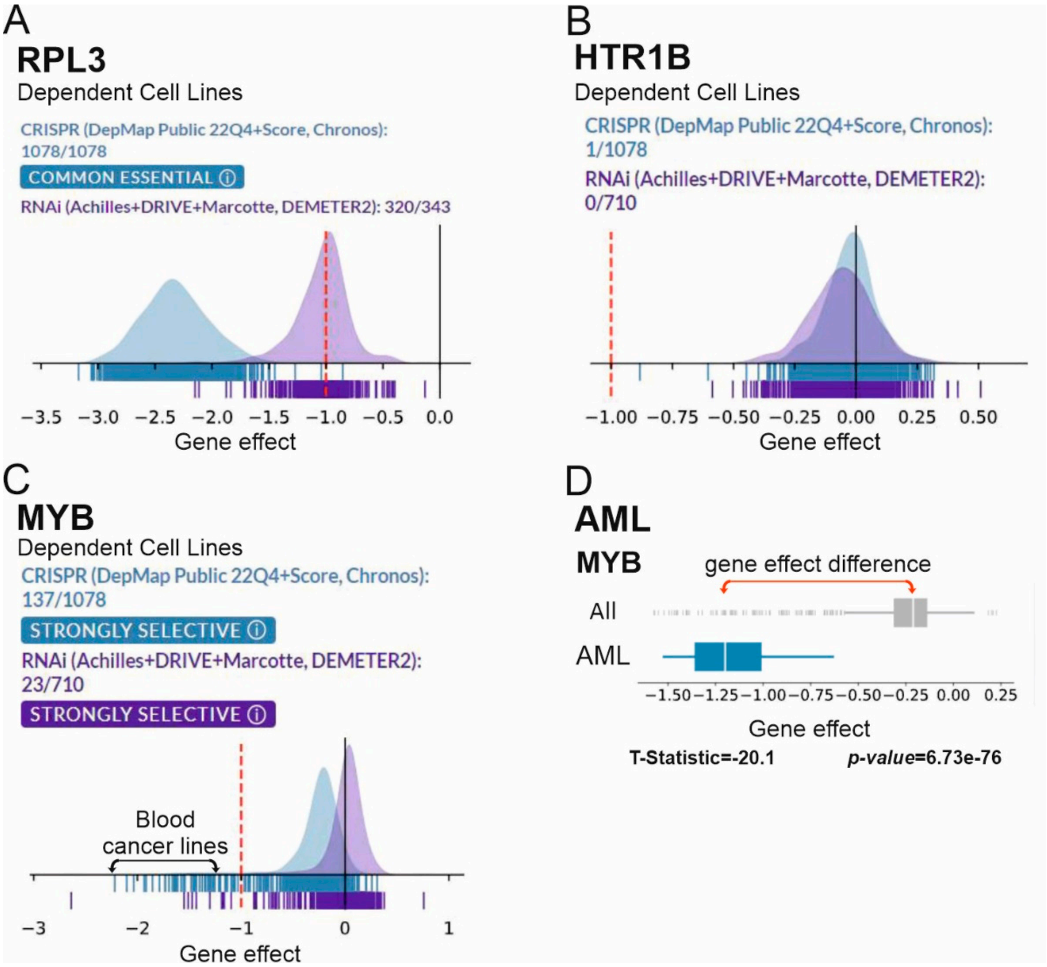

Supplementary Figure S1. Gene effects upon CRISPR/Cas9 deletion or RNAi down-regulation for the *RPL3* (A), *HTR1B* (B) and *MYB* (C) genes. (D) Example of gene effect difference (red bracket) that can be calculated as a T-statistic parameter. See text for details.

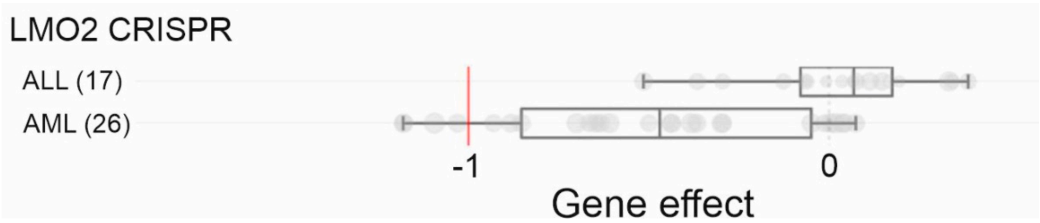

Supplementary Figure S2. Gene effects of *LMO2* knockout by CRISPR/Cas9 in AML (17 cell lines) and ALL (26 cell lines). Gray circles indicate the value of 'Gene effect' upon deletion of the *LMO2* gene for each cell line.

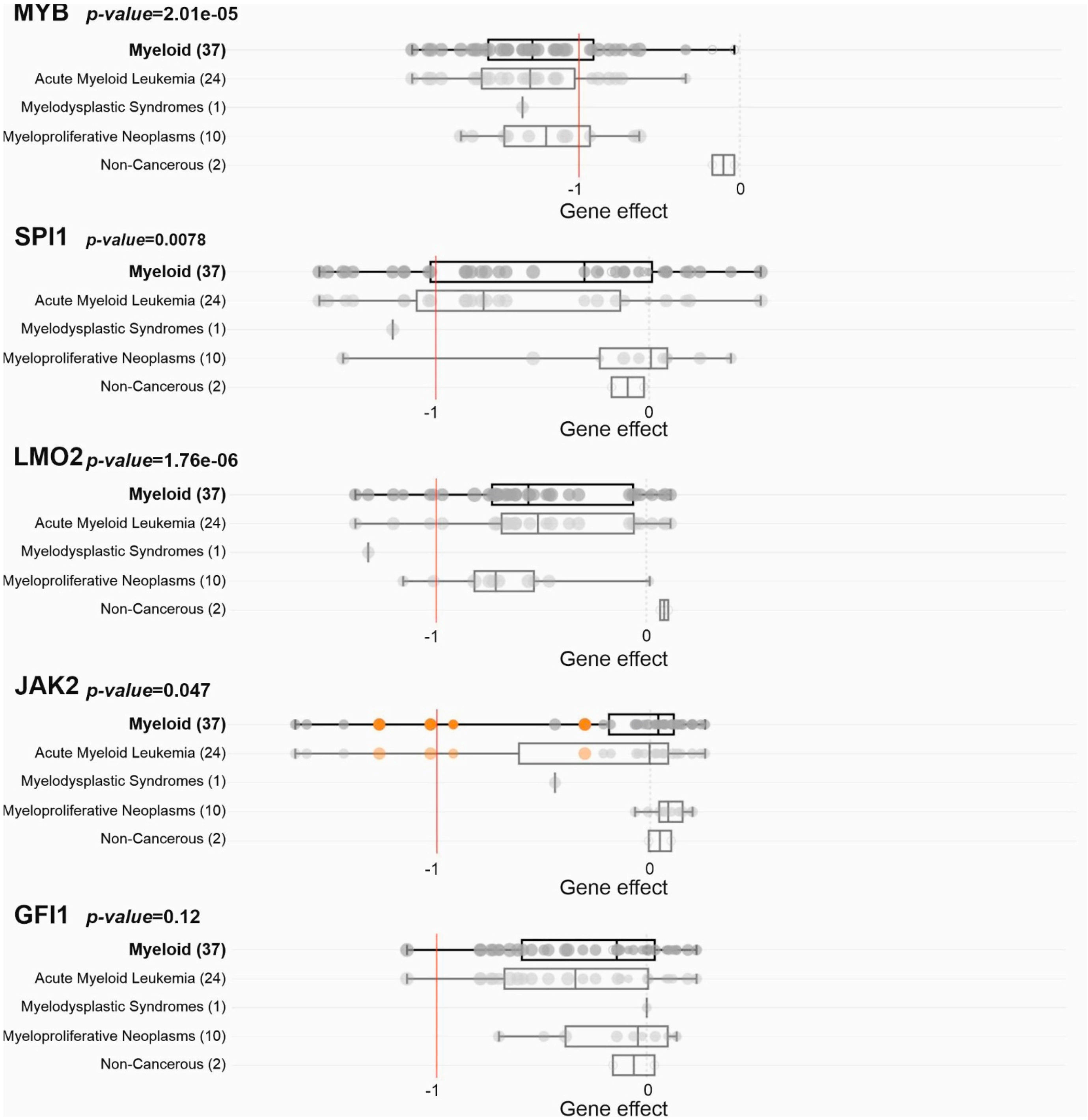

Supplementary Figure S3. Gene effects in myeloid cell lines upon CRISPR/Cas9 deletion of AML Supertargets.

Shown are transformed (AML) and non-malignant cell lines. Circles indicate the value of 'Gene effect' upon deletion of the Supertargets for each cell line. Grey circles indicate cell lines with the wild type Supertarget gene. Orange, cell lines with mutant Supertarget gene.

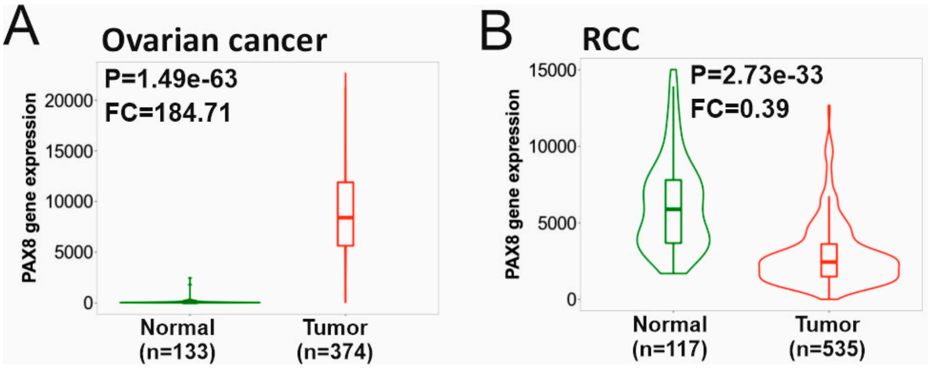

Supplementary Figure S4. Differential expression of the *PAX8* gene in normal (green) *vs* tumor (red) samples in ovarian cancer and RCC (presented as violin plots). P, p-value, Mann-Whitney test. FC, fold change median.

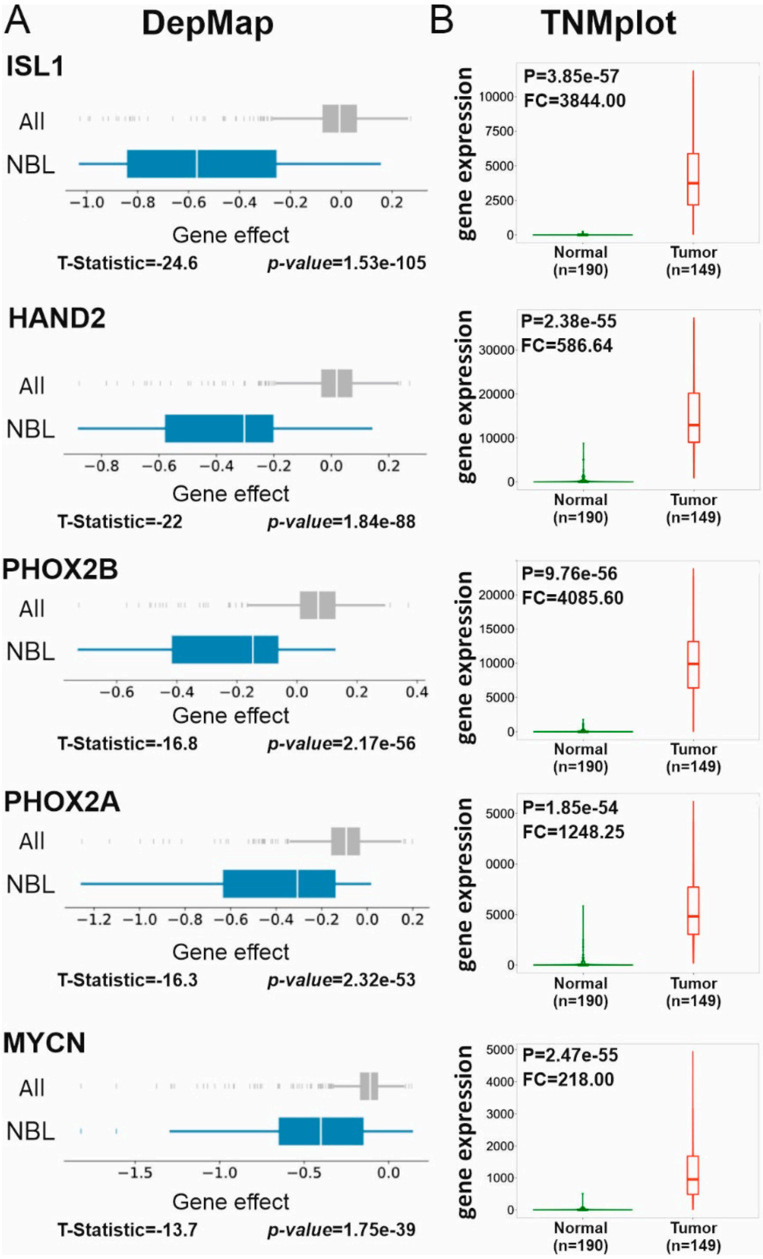

Supplementary Figure S5. Supertarget genes in neuroblastoma (31 cell lines).

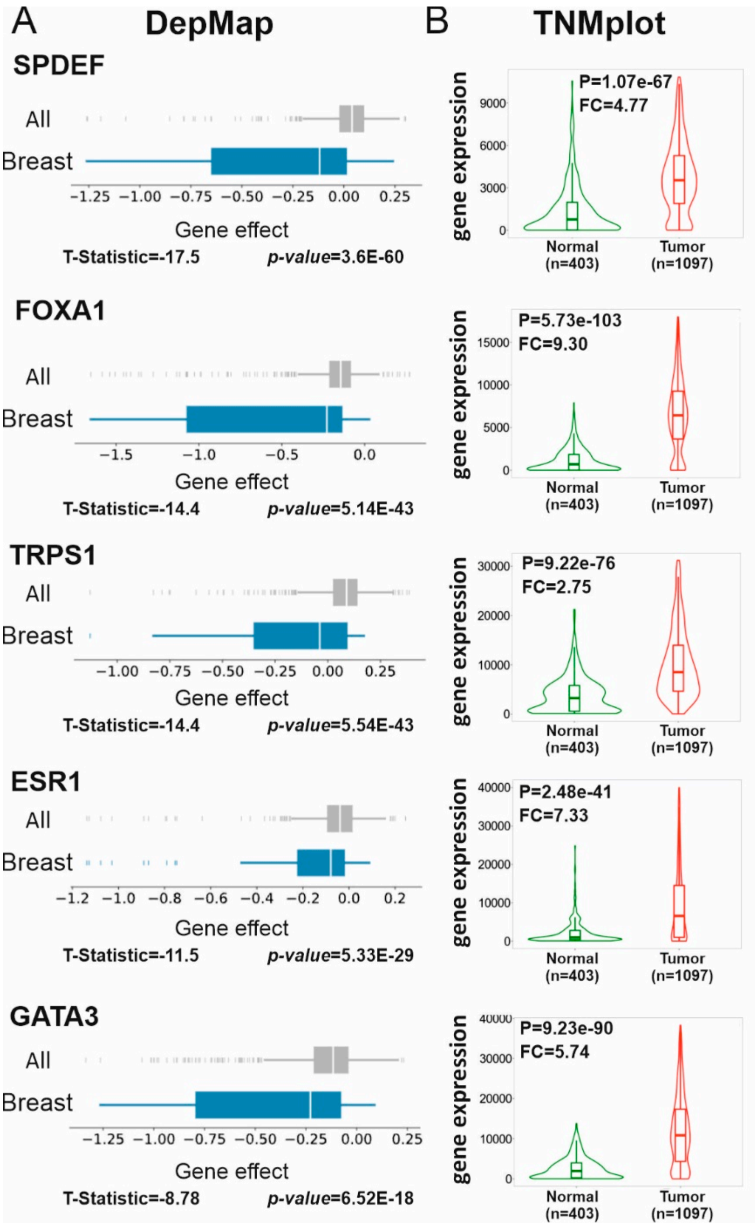

Supplementary Figure S6. Supertarget genes in breast cancer (45 cell lines).

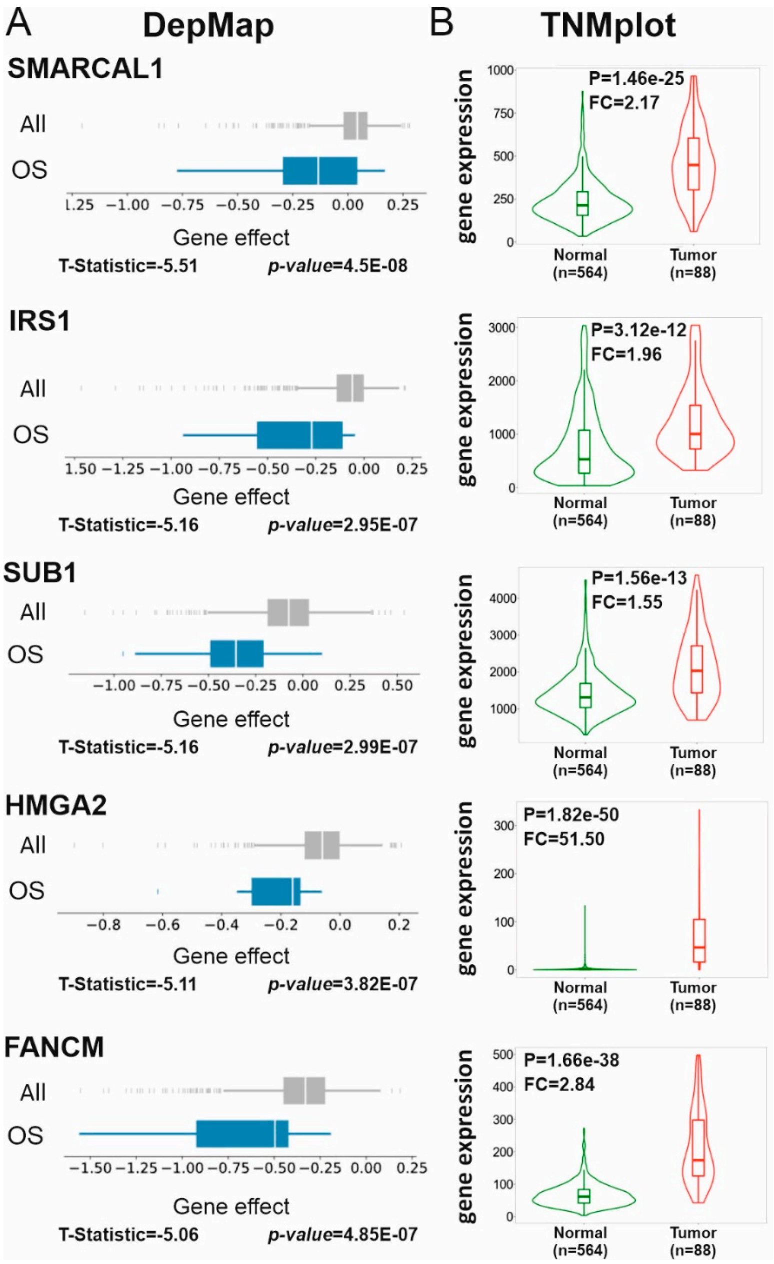

Supplementary Figure S7. Supertarget genes in osteosarcoma (9 cell lines).

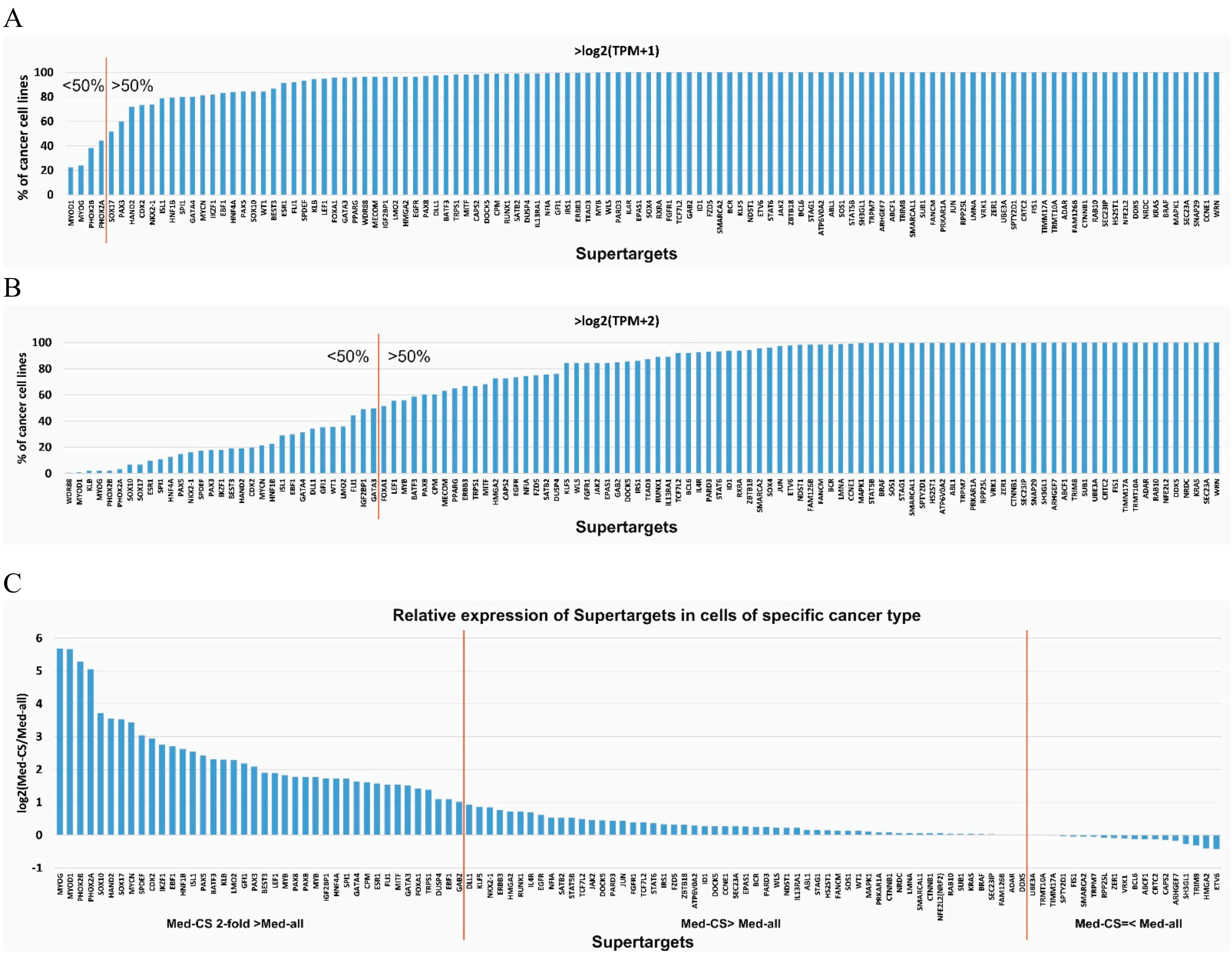

Supplementary Figure S8. Expression of Supertarget genes.

(A) Percentages of tumor cell lines (total 1,408) in which Supertargets are expressed > $\log_2$(TPM+1). (B) Percentages of tumor cell lines in which Supertargets are expressed > $\log_2$(TPM+2). (C) Relative expression ($\log_2$(Med-CS/Med-all) of Supertargets. Med-CS (cancer specific), median expression in tumor cell line where the Supertarget was initially identified. Med-all, median expression of Supertargets in all tumor cell lines.

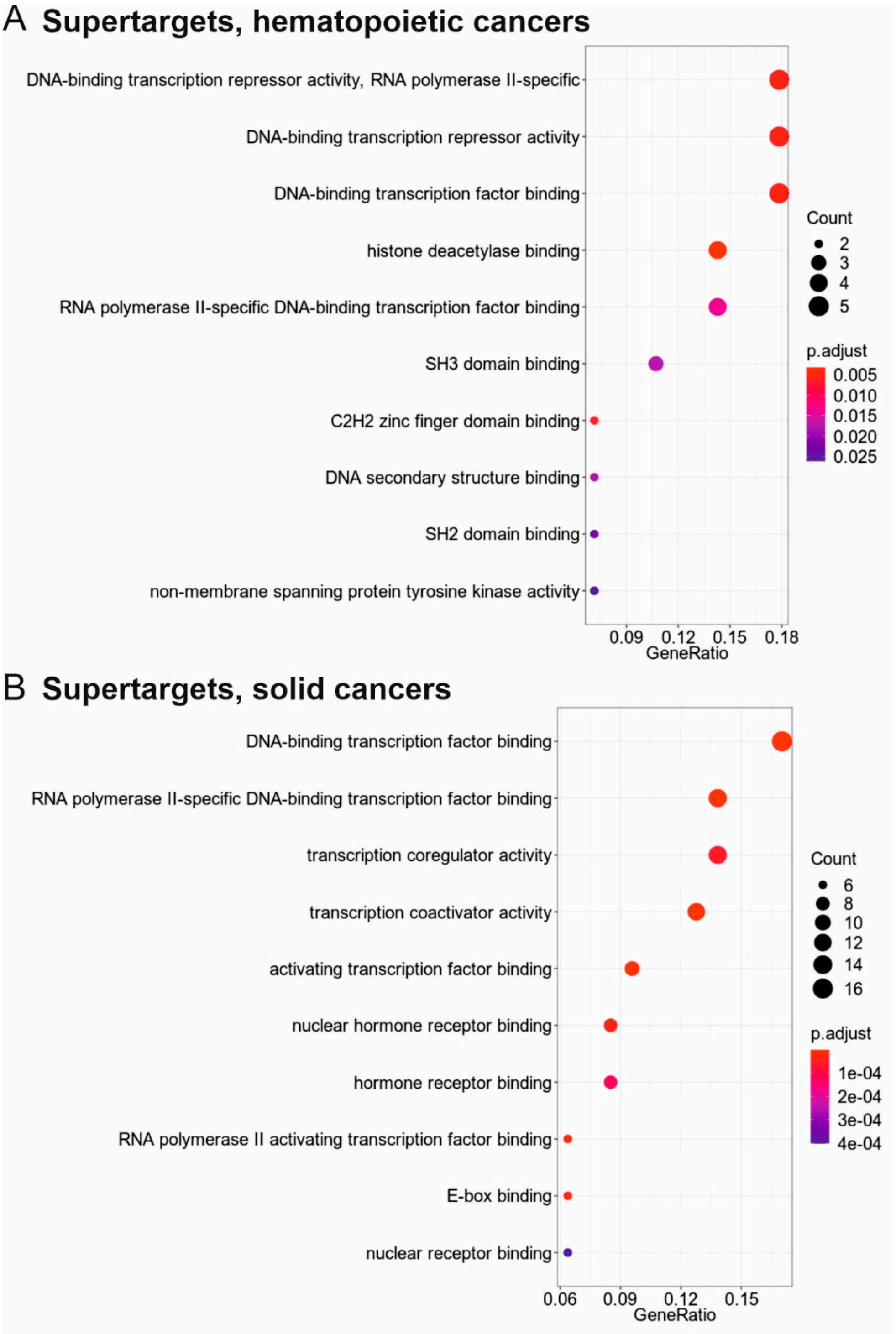

Supplementary Figure S9. GOplot: molecular function (TNMplot resource).

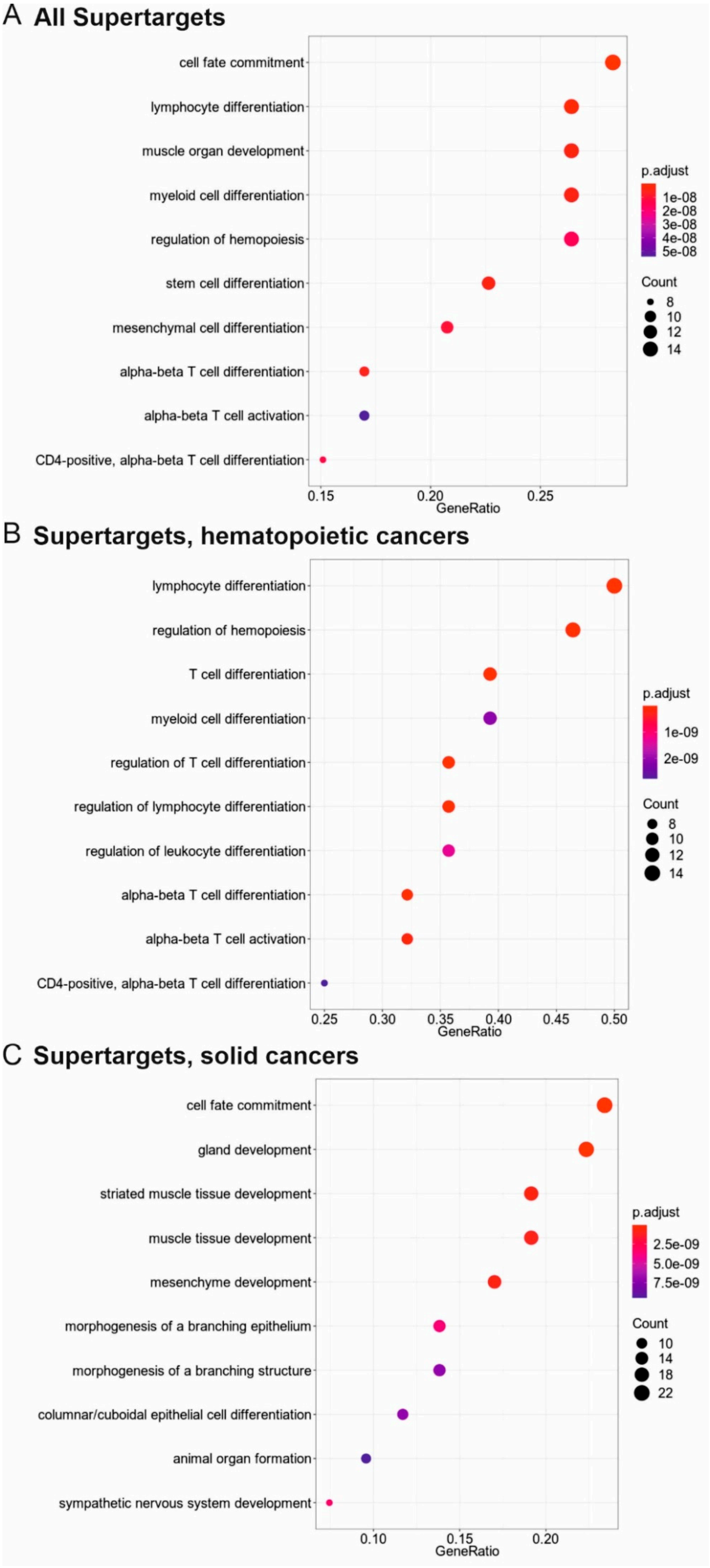

Supplementary Figure S10. GOplot: biological pathways (TNMplot resource).

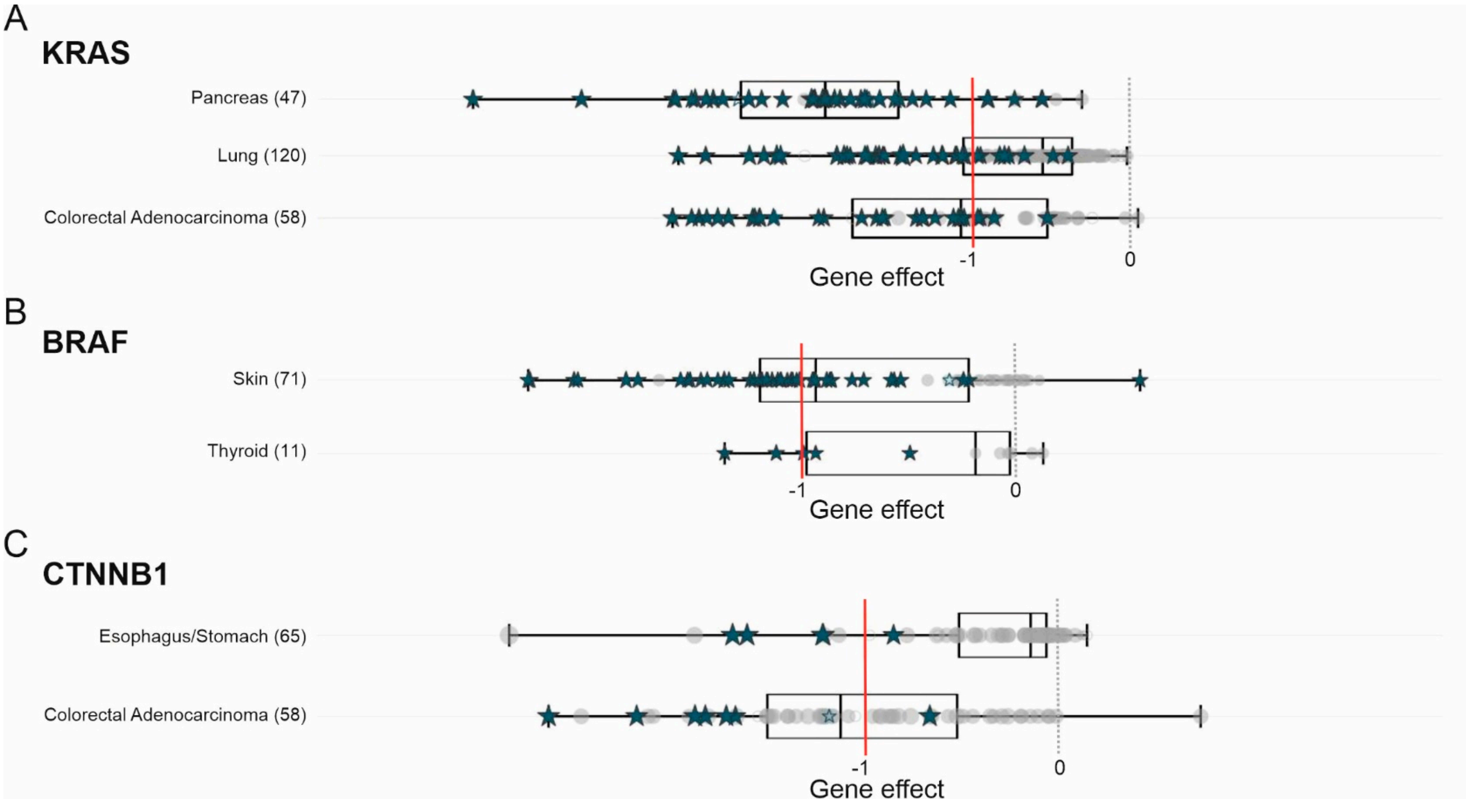

Supplementary Figure S11. Driver mutations in *KRAS* (A), *BRAF* (B) and *CTNNB1* (C) Supertarget genes. Grey circles, cell lines with the wild type Supertarget gene; blue asterisks, cell lines with the mutant Supertarget.
