# Supplementary material for: Analyses of Genes Critical to Tumor Survival Reveal Potential ‘Supertargets’: Focus on Transcription
Source: Cancers (Basel). 2023 Jun 3;15(11):3042. doi: 10.3390/cancers15113042 (PMC10252933; doi:10.3390/cancers15113042)
Supplement: Supplementary file 1 [file cancers-15-03042-s001.zip › cancers-2405642-Supplementary File S3. GO analysis.pdf]

Analysis Type:	PANTHER Overrepr
Annotation Version and Release Date:	PANTHER version 1
Analyzed List:	upload_1 (Homo sa
Reference List:	Homo sapiens (all g
Test Type:	FISHER
Correction:	FDR
PANTHER GO-Slim Molecular Function	Homo sapiens - REF
fibroblast growth factor binding (GO:0017134)	6
growth factor binding (GO:0019838)	35
DNA-binding transcription activator activity, RNA polymerase II-specific (GO:0001228)	38
DNA-binding transcription activator activity (GO:0001216)	38
transcription factor binding (GO:0008134)	63
protein tyrosine kinase activity (GO:0004713)	96
cis-regulatory region sequence-specific DNA binding (GO:0000987)	810
RNA polymerase II cis-regulatory region sequence-specific DNA binding (GO:0000978)	798
RNA polymerase II transcription regulatory region sequence-specific DNA binding (GO:00	1059
DNA-binding transcription factor activity, RNA polymerase II-specific (GO:0000981)	984
DNA-binding transcription factor activity (GO:0003700)	1054
transcription cis-regulatory region binding (GO:0000976)	1099
transcription regulatory region nucleic acid binding (GO:0001067)	1099
sequence-specific double-stranded DNA binding (GO:1990837)	1117
sequence-specific DNA binding (GO:0043565)	1148
double-stranded DNA binding (GO:0003690)	1164
transcription regulator activity (GO:0140110)	1265
DNA binding (GO:0003677)	1361
nucleic acid binding (GO:0003676)	1974
heterocyclic compound binding (GO:1901363)	2375
organic cyclic compound binding (GO:0097159)	2412
binding (GO:0005488)	5893
molecular_function (GO:0003674)	9609
Unclassified (UNCLASSIFIED)	10980

esentation Test (Released 20221013)
7.0 Released 2022-02-22
piens)
;enes in database)

upload_1 (	upload_1 (	upload_1 (	upload_1 (	upload_1 (	upload_1 (FDR)
2	.04	+	54.47	1.00E-03	2.49E-02
4	.21	+	18.67	9.10E-05	2.62E-03
3	.23	+	12.90	1.98E-03	4.70E-02
3	.23	+	12.90	1.98E-03	4.50E-02
4	.39	+	10.37	7.44E-04	1.93E-02
5	.59	+	8.51	3.82E-04	1.04E-02
42	4.96	+	8.47	4.45E-27	2.21E-25
41	4.88	+	8.40	2.98E-26	1.35E-24
52	6.48	+	8.02	5.26E-33	2.87E-30
48	6.02	+	7.97	4.21E-30	2.55E-28
51	6.45	+	7.91	5.19E-32	7.09E-30
52	6.73	+	7.73	2.98E-32	8.13E-30
52	6.73	+	7.73	2.98E-32	5.42E-30
52	6.84	+	7.61	6.36E-32	6.94E-30
52	7.03	+	7.40	2.28E-31	1.78E-29
52	7.12	+	7.30	4.34E-31	2.96E-29
54	7.74	+	6.98	1.78E-31	1.62E-29
54	8.33	+	6.48	6.01E-30	3.28E-28
58	12.08	+	4.80	7.72E-26	3.24E-24
62	14.53	+	4.27	3.23E-25	1.26E-23
62	14.76	+	4.20	7.33E-25	2.67E-23
84	36.06	+	2.33	1.57E-18	5.34E-17
95	58.80	+	1.62	7.04E-11	2.14E-09
31	67.20	-	.46	7.04E-11	2.26E-09
