# Supplementary material for: Analyses of Genes Critical to Tumor Survival Reveal Potential ‘Supertargets’: Focus on Transcription
Source: Cancers (Basel). 2023 Jun 3;15(11):3042. doi: 10.3390/cancers15113042 (PMC10252933; doi:10.3390/cancers15113042)
Supplement: Supplementary file 1 [file cancers-15-03042-s001.zip › cancers-2405642-Supplementary File S4. DNA-binding domains of Supertargets.pdf]

Acordingly HOmo sapiens COmprehensive MOdel COllection (HOCOMOCO) v11, PMID: 29140464

Superclass

Basic domains

Zinc-coordinating DNA-binding domains

Helix-turn-helix domains

Other all-alpha-helical DNA-binding domains

alpha-Helices exposed by beta-structures

Immunoglobulin fold

beta-Hairpin exposed by an alpha/beta-scaffold

beta-Sheet binding to DNA

beta-Barrel DNA-binding domains

Yet undefined DNA-binding domains

Class

Basic leucine zipper factors (bZIP)

Basic helix-loop-helix factors (bHLH)

Basic helix-span-helix factors (bHSH)

Nuclear receptors with C4 zinc fingers

Other C4 zinc finger-type factors

C2H2 zinc finger factors

DM-type intertwined zinc finger factors

CXXC zinc finger factors

C2HC zinc finger factors

C3H zinc finger factors

C2CH THAP-type zinc finger factors

Homeo domain factors

Paired box factors

Fork head / winged helix factors

Heat shock factors

Tryptophan cluster factors

TEA domain factors

ARID domain factors

High mobility group (HMG) domain

Heteromeric CCAAT-binding factors

MADS box factors

SAND domain factors

Rel homology region (RHR) factors

STAT domain factors

p53 domain factors

Runt domain factors

T-Box factors

NDT80 domain factors

Grainyhead domain factors

SMAD/NF-1 DNA-binding domain factors

GCM domain factors

TATA-binding proteins

A.T hook factors

Cold shock domain factors

Yet undefined DNA binding domains

Number in human genome
58
111
5
48
19
697
8
7
12
6
12
244
9
68
6

68

4

15

44

3

5

8

25

7

3

3

17

1

6

12

3

2

3

19

Number in Supertargets
3
5
0
3
2
9
0
0
0
0
0
7
3
1
0

6

1

0

5

0

2

0

2

0

1

1

0

0

0

1

0

1

0

0

Supertargets/Genome, %
5.2%
4.5%
0.0%
6.3%
10.5%
1.3%
0.0%
0.0%
0.0%
0.0%
0.0%
2.9%
33.3%
1.5%
0.0%

8.8%

25.0%

0.0%

11.4%

0.0%

40.0%

0.0%

8.0%

0.0%

33.3%

33.3%

0.0%

0.0%

0.0%

8.3%

0.0%

50.0%

0.0%

0.0%
